# Normal triglyceride concentration and the risk of diabetes mellitus type 2 in the general population of China

**DOI:** 10.3389/fendo.2024.1330650

**Published:** 2024-02-08

**Authors:** Rubing Guo, Lianhua Wei, Yongtong Cao, Wei Zhao

**Affiliations:** ^1^ School of Public Health, Gansu University of Traditional Chinese Medicine, Lanzhou, China; ^2^ Department of Clinical Laboratory, Gansu Provincial Hospital, Lanzhou, China; ^3^ Department of Clinical Laboratory, China-Japan Friendship Hospital, Beijing, China

**Keywords:** triglycerides, diabetes, cohort study, dyslipidemia, survival analysis

## Abstract

**Introduction:**

Hypertriglyceridemia and its derivatives are independent predictors of diabetes mellitus type 2 (T2DM). However, the relationship between triglyceride concentrations within the normal range and the incidence of T2DM remains to be clarified. This study investigated the potential relationship between variations in plasma triglyceride levels within the normal range and T2DM onset using data from a longitudinal study of health and retirement in China.

**Methods:**

Between, 2010 and, 2016, we conducted a retrospective cohort study involving 36,441 individuals with normal triglyceride levels. Using a Cox proportional hazards regression model, we examined the connection between normal triglyceride levels and T2DM incidence. We employed this method with smooth curve fitting to investigate potential nonlinear associations. Subgroup analyses were performed based on age, sex, body mass index, smoking and drinking status, hypertension, and family history of diabetes.

**Results:**

A significant linear relationship was observed between normal triglyceride levels and the incidence of T2DM. The hazard ratio for T2DM in individuals with normal triglycerides was 1.81 (95% confidence interval: 1.39, 2.36); P<0.001). Kaplan–Meier analysis further demonstrated a prospective association between the higher tertiles of normal triglyceride levels and the development of T2DM (P<0.001). Subgroup analysis revealed a stronger positive correlation between normal triglyceride levels in females and the risk of T2DM.

**Discussion:**

An increase in triglyceride levels within the normal range is related to a continuous increase in the incidence of T2DM in the general population. These findings show that almost everyone can benefit from reducing triglyceride levels, further emphasizing the importance of lifestyle changes in the general population.

## 1 Introduction

Diabetes is a complex metabolic disorder influenced by both genetic and environmental factors. It is characterized by reduced insulin sensitivity, inadequate insulin production, and impaired biological functions. The global prevalence of diabetes is steadily increasing, posing a significant public health challenge. By, 2021, 536.6 million adults aged 20–79 years worldwide will have diabetes (uncertainty interval: 424.2–612.3 million), and it is estimated that 783.2 million people will have diabetes by, 2045 (uncertainty interval: 605.2–898.6 million) (1). Worryingly, nearly half of the world’s adults (44.7%; 239.7 million) do not know that they have diabetes. China has the highest number of patients with undiagnosed diabetes (2).

Triglycerides are lipids in the bloodstream that are a significant source of energy storage and supply within the human body. Elevated triglyceride levels have been linked to an increased risk of cardiovascular diseases, including coronary heart disease, heart failure, and atherosclerosis (3–7). Detecting triglyceride levels can help assess patients’ cardiovascular risk, and take corresponding preventive measures. Regarding diabetes, elevated triglyceride levels increase the risk of diabetes and prediabetes (8, 9). An increasing number of studies have found that triglycerides combined with other indicators as a composite indicator (such as the triglyceride glucose index) are an independent predictor of diabetes (10–12). In addition, research suggests that high triglyceride levels increase the risk of insulin resistance, as they can lead to fat accumulation in the liver and muscles. This accumulation triggers insulin resistance and affects the secretion of adipokines, thereby further impacting insulin signaling and glucose uptake (13). This close association between triglyceride levels and the development of diabetes highlights the importance of triglycerides as a biomarker for assessing the risk of diabetes.

Nevertheless, there are limited reports or cohort studies that have corroborated the association between normal triglyceride levels and the onset of diabetes mellitus type 2 (T2DM) following thorough adjustment for possible confounding factors. Furthermore, the exact dose-response correlation between gradual fluctuations in triglyceride levels within the normal range and the development of T2DM is still uncertain. Consequently, this study investigated the potential relationship between variations in plasma triglyceride levels within the normal range and T2DM onset using data from a longitudinal study of health and retirement in China. This study included a sample of 36,441 participants from 32 locations in 11 cities in China. The aim of this study was to investigate the relationship between normal triglyceride levels and the risk of developing T2MD, and to describe the related dose-response relationship.

## 2 Methods

### 2.1 Data source

The dataset used in this study was sourced from the DATADRYAD database (www.datadryad.org) that was originally published by Chen et al. The data for this study were derived from the primary research data of the article titled “Association of body mass index and age with incident diabetes in Chinese adults” (14) (Dryad, https://doi.org/10.5061/dryad.ft8750v). This work is licensed under a CC0 1.0 Universal (CC0 1.0) Public Domain Dedication license.

### 2.2 Study population

**Figure 1:**
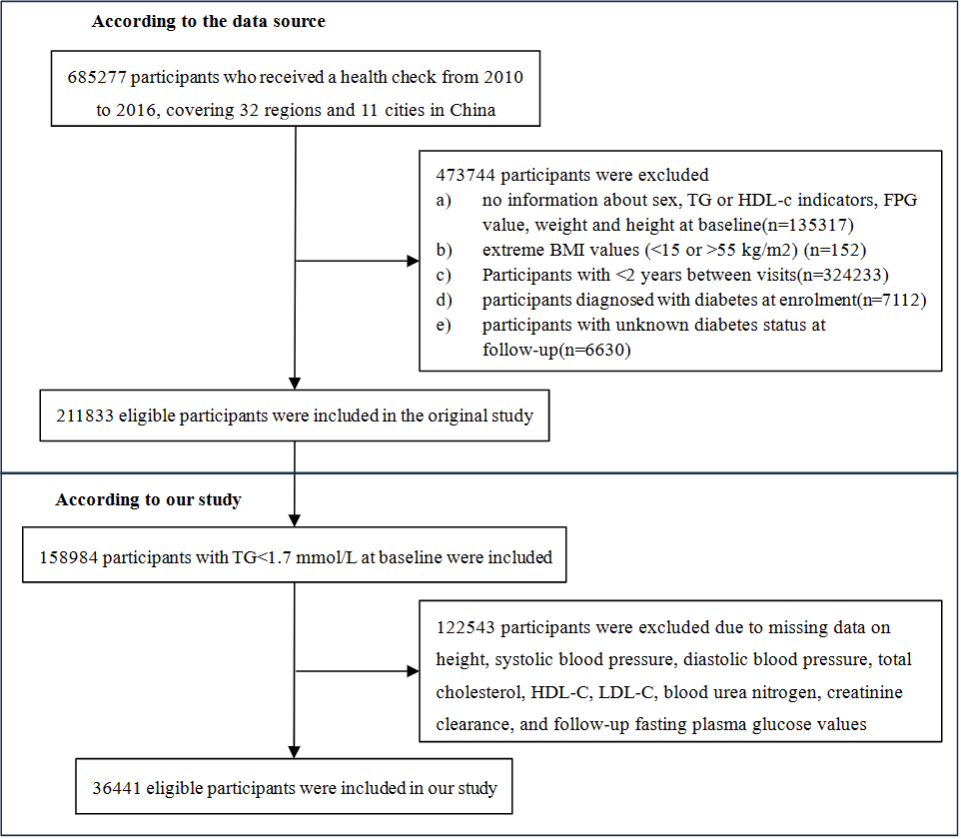
Study design and flowchart of study participants.

The primary data used in this study were acquired from a computerized database established by the Rich Healthcare Group in China. The comprehensive database used in this study included medical records of all participants who underwent health examinations at 32 sites across 11 major Chinese cities. The database used in this study encompassed 685,277 study participants aged ≥20 years who had a minimum of two visits between, 2010 and, 2016. Exclusion criteria were applied to ensure data quality and integrity. Specifically, participants were excluded if they had missing baseline weight, height, sex, extreme body mass index (BMI) values (<15 kg/m^2^ or >55 kg/m^2^), or no available fasting plasma glucose (FPG) values (n=103,946, n=1, n=152, n=31,370, respectively). Additionally, individuals with a baseline diagnosis of diabetes (2,997 participants with self-reported diagnosis and 4,115 participants diagnosed by FPG≥7.0 mmol/L), a follow-up interval of less than 2 years (n=324,233), or unclear diabetes status at follow-up (n=6,630) were also excluded. Consequently, the final study cohort comprised 211,833 participants. According to the National Cholesterol Education Program (NCEP) guidelines, 158,984 participants with baseline triglycerides (TG)<1.7 mmol/L were included in our study. After applying strict exclusion criteria, 122,543 participants were excluded from the initial dataset due to missing baseline measurements of height, systolic blood pressure (SBP), diastolic blood pressure (DBP), cholesterol, high-density lipoprotein cholesterol (HDL-C), low-density lipoprotein cholesterol (LDL-C), blood urea nitrogen (BUN), creatinine clearance rate (CCR), and follow-up FPG values. Thus, a final cohort of 36,441 eligible participants was used for comprehensive data analysis. The flowchart in **Figure 1** visually represents the participant selection process.

### 2.3 Data collection

Classification variables, including sex, smoking status, drinking status, and family history of diabetes, were collected from all participants. Continuous variables were also recorded, such as age, BMI, SBP, DBP, cholesterol, HDL-C, LDL-C, alanine aminotransferase (ALT), aspartate aminotransferase (AST), and FPG. At each visit to the health examination center, detailed information on demographic characteristics, such as age and sex, and lifestyle habits, including smoking and alcohol consumption, were collected through comprehensive questionnaires. Additionally, the participants were asked about their family history of diabetes. Height, weight, and blood pressure were measured by trained staff using standardized protocols. BMI (kg/m^2^) was calculated by dividing the weight (in kilograms) by the square of height (in meters). An automated analyzer (Beckman, 5800) was used to conduct laboratory tests for cholesterol, triglyceride, LDL-C, and HDL-C levels. Blood samples were collected from fasting individuals after a minimum of 10 hours of fasting. The glucose oxidase method was used to measure plasma glucose levels using the same automated analyzer (Beckman, 5800).

### 2.4 Definition of diabetes mellitus type 2

T2DM was diagnosed using one or more of the following established criteria, which have been previously validated: fasting blood glucose levels of 7.0 mmol/L or higher, and self-reported T2DM during the follow-up period (15).

### 2.5 Statistical analysis

Normally distributed data are presented as mean ± standard deviation for continuous variables, whereas the median (quartile) is used for data with skewed distribution. Categorical variables are presented as numbers (percentages). To analyze the baseline characteristics of the different triglyceride tertiles, one-way ANOVA was employed for continuous variables with normal distribution, the Kruskal–Wallis H test for those with skewed distribution, and the chi-square test for categorical variables. A multivariate Cox regression analysis was conducted to assess the risk of developing T2DM, yielding the hazard ratio (HR) and 95% confidence interval (95% CI). The unadjusted and adjusted multivariable models are listed. In cases where the exclusion of covariates in the complete model leads to a change exceeding 10% in the regression coefficient for normal triglyceride concentration or if the p-value for the covariate regression coefficient on the incidence of T2DM is below 0.1, the covariate is deemed to have an impact and requires adjustment. In this study, the Cox model was adjusted for age, sex, BMI, DBP, FPG, cholesterol, HDL-C, LDL-C, AST, ALT, BUN, CCR, smoking status, drinking status, hypertension, and family history of diabetes. The Kaplan–Meier curve was constructed based on the triglyceride tertiles, and the significance was discussed using the log-rank test. A generalized additive model incorporating a smoothing function was used to explore the potential nonlinear association between triglycerides and the occurrence of T2DM. For subgroup analysis, a hierarchical Cox regression model was used. Interactions between the subgroups were evaluated using the likelihood ratio test. Statistical analyses were performed using the R software package (http://www.r-project.org, R Foundation), SPSS (version 26.0; SPSS Inc., Chicago, Illinois, USA), and EmpowerStats (www.empowerstats.net, X&Y Solutions, Inc., Boston, Massachusetts). Statistical significance was set at P<0.05 (two-tailed).

## 3 Results

### 3.1 Baseline characteristics of individuals

This study included 34,441 individuals, with a mean age of 42.76 years. Among them, 50.63% were male and 49.37% were female. **Table 1** presents the baseline characteristics of the participants according to the tertiles of fasting triglyceride levels. Participants with higher fasting triglyceride levels were more likely to be male, older, and have higher BMI, DBP, SBP, FPG, AST, ALT, LDL-C, BUN, and CCR. They were also more likely to have hypertension, drink, smoke, and have lower HDL-C levels (all P<0.05). However, the participants were healthy, and the recorded values were generally within the normal range. Moreover, no significant difference was observed in the proportion of individuals with a family history of diabetes among the tertiles of fasting triglyceride levels (P=0.497).

**Table 1:** Baseline characteristics stratified by tertiles of fasting triglyceride levels.

Variable	Low tertile (n=12114)	Medium tertile (n=11280)	High tertile (n=13047)	P value
Triglyceride (mmol/L)	0.6 ± 0.1	0.9 ± 0.1	1.3 ± 0.2	<0.001
Age (years)	39.8 ± 11.3	42.7 ± 12.7	45.6 ± 13.4	<0.001
Sex				<0.001
Male	4116 (34.0%)	5638 (50.0%)	8237 (63.1%)	
Female	7998 (66.0%)	5642 (50.0%)	4810 (36.9%)	
BMI (kg/m2)	21.6 ± 2.7	22.6 ± 3.0	23.9 ± 3.1	<0.001
SBP (mmHg)	113.7 ± 15.0	117.2 ± 15.9	121.7 ± 16.6	<0.001
DBP (mmHg)	70.4 ± 9.9	72.9 ± 10.3	75.6 ± 10.8	<0.001
FPG (mmol/L)	4.9 ± 0.5	4.9 ± 0.6	5.0 ± 0.6	<0.001
Cholesterol (mmol/L)	4.4 ± 0.7	4.6 ± 0.8	4.9 ± 0.9	<0.001
HDL-C (mmol/L)	1.5 ± 0.3	1.4 ± 0.3	1.4 ± 0.3	<0.001
LDL-C (mmol/L)	2.5 ± 0.6	2.7 ± 0.6	2.9 ± 0.7	<0.001
ALT (U/L)	14.0 (11.0-19.0)	16.2 (12.0-23.1)	20.0 (14.1-28.7)	<0.001
AST (U/L)	20.0 (17.1-24.0)	21.0 (18.0-25.0)	22.5 (19.0-27.0)	<0.001
BUN (mmol/L)	4.6 ± 1.2	4.6 ± 1.2	4.7 ± 1.2	<0.001
CCR (umol/L)	66.6 ± 14.9	70.4 ± 15.6	73.8 ± 16.8	<0.001
Family history of diabetes	242 (2.0%)	228 (2.0%)	286 (2.2%)	0.497
Hypertension	902 (7.45%)	1275 (11.30%)	2271 (17.41%)	<0.001
Smoking status				<0.001
Yes	346 (2.86%)	537 (4.76%)	908 (6.96%)	
No	2485 (20.5%)	2320 (20.6%)	2521 (19.3%)	
Not recorded	9283 (76.6%)	8423 (74.7%)	9618 (73.7%)	
drinking status				<0.001
Yes	435 (3.6%)	558 (5.0%)	830 (6.4%)	
No	2396 (19.8%)	2299 (20.4%)	2599 (19.9%)	
Not recorded	9283 (76.6%)	8423 (74.7%)	9618 (73.7%)	

Continuous variables with a normal distribution were reported as mean ± standard deviation (SD), whereas skewed continuous variables were presented as medians (interquartile range [IQR]). Categorical data are presented as n (%). To assess the presence of statistically significant differences among the tertiles, the χ2-test was employed for categorical variables, the Kruskal–Wallis test was used for skewed variables, and one-way analysis of variance (ANOVA) was applied for variables with a normal distribution.

ALT, alanine aminotransferase; AST, aspartate aminotransferase; BMI, body mass index; BUN, blood urea nitrogen; CCR, creatinine clearance rate; DBP, diastolic blood pressure; FPG, fasting plasma glucose; HDL-C, high-density lipoprotein cholesterol; LDL-C, low-density lipoprotein cholesterol; SBP, systolic blood pressure.

### 3.2 Incidence of diabetes mellitus type 2

**Figure 2:**
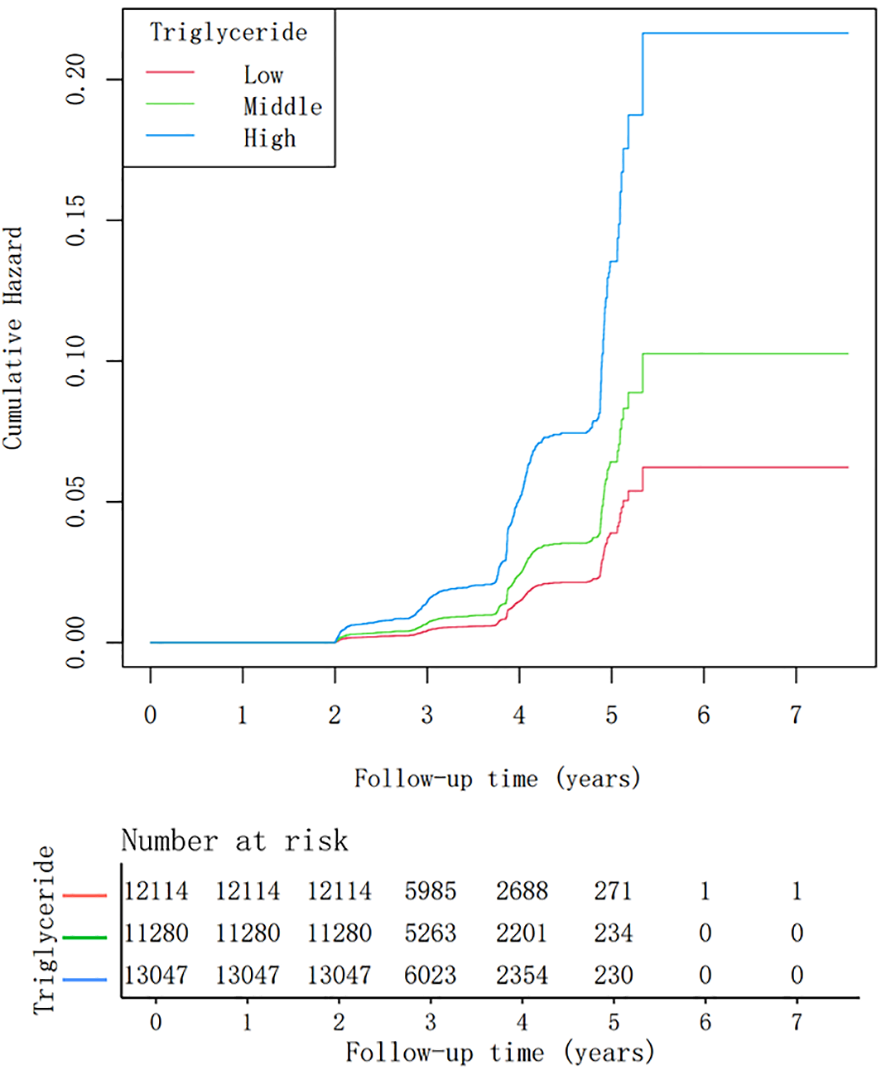
Kaplan-Meier analysis of diabetes mellitus type 2 incidence stratified by tertiles of triglyceride levels (log-rank test, P< 0.001).

During a mean follow-up period of 3.09 years, 593 (1.63%) individuals developed T2DM. The cumulative incidence of T2DM stratified by tertiles of fasting triglyceride levels is shown in **Figure 2**. Kaplan–Meier analysis revealed a significant association between higher triglyceride tertiles and an increased incidence of T2DM (P<0.001).

### 3.3 Association of normal triglyceride levels with diabetes mellitus type 2 risk

The association between triglyceride concentration and the risk of T2DM was assessed using multivariate Cox regression analysis (**Table 2**). The results of the three Cox regression models adjusted to varying degrees consistently demonstrated similar key findings. In the crude model, triglyceride levels were positively correlated with the risk of diabetes mellitus type 2 [HR and 95% CI=4.76 (3.77, 6.01), P<0.001]. In the fully adjusted model III, the HR value weakened, but a positive correlation between triglyceride concentration and the risk of T2DM persisted [HR and 95% CI=1.81 (1.39, 2.36), P<0.001]. Sensitivity analysis was performed by treating triglyceride concentration as a categorical variable. In the fully adjusted model, the HR and 95% CI for the middle and high tertiles of triglyceride concentration were 1.12 (0.87, 1.45) and 1.51 (1.19, 1.92), respectively (P for trend<0.001).

**Table 2:** Association of normal triglyceride levels with incident diabetes mellitus type 2.

Outcome	Crude Model		Model I		Model II		Model III	
	HR (95% CI)	P-value	HR (95% CI)	P-value	HR (95% CI)	P-value	HR (95% CI)	P-value
Triglyceride(mmol/L)	4.76 (3.77, 6.01)	<0.001	2.70 (2.11, 3.45)	<0.001	1.79 (1.39, 2.31)	<0.001	1.81 (1.39, 2.36)	<0.001
Triglyceride(mmol/L) tertile								
Low	Reference		Reference		Reference		Reference	
Middle	1.65 (1.28, 2.12)	<0.001	1.19 (0.92, 1.54)	0.179	0.95 (0.73, 1.23)	0.705	1.12 (0.87, 1.45)	0.386
High	3.48 (2.79, 4.34)	<0.001	2.01 (1.60, 2.52)	<0.001	1.40 (1.11, 1.77)	0.004	1.51 (1.19, 1.92)	<0.001
P for trend	<0.001		<0.001		< 0.001		<0.001	

Model I was adjusted for age and sex.

Model II was adjusted for age, sex, BMI, SBP, DBP, hypertension, smoking status, drinking status, and family history of diabetes.

Model III was adjusted for age, sex, BMI, SBP, DBP, FPG, cholesterol, HDL-C, LDL-C, ALT, AST, BUN, CCR, hypertension, smoking status, drinking status, and family history of diabetes.

CI, confidence interval; HR, hazard ratios; other abbreviations as in **Table 1**.

### 3.4 Threshold effect analysis of normal triglyceride levels on incident diabetes mellitus type 2

**Figure 3:**
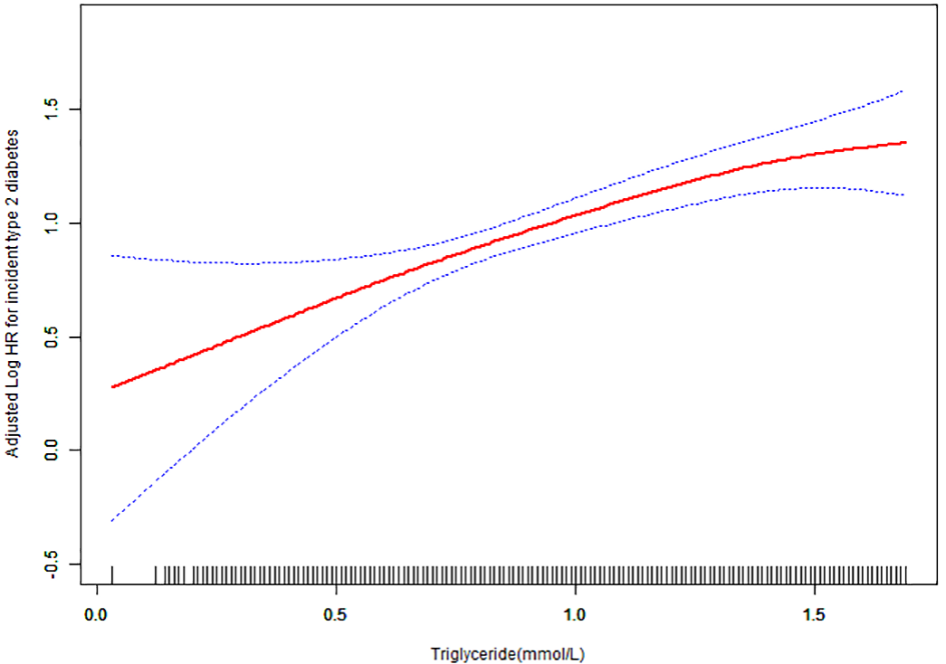
Hazard ratios (95% confidence intervals) for the association between triglyceride concentration and the risk of diabetes mellitus type 2. The red line represents the estimated risk of diabetes mellitus type 2, whereas the blue line represents the 95% confidence interval after adjusting for covariates.

We employed a smoothing curve function analysis to evaluate the association between normal triglyceride concentrations and the risk of T2DM (**Figure 3**). This analysis showed that after adjusting for age, sex, BMI, DBP, FPG, cholesterol, HDL-C, LDL-C, AST, ALT, BUN, CCR, smoking status, and family history of diabetes, there was a continuous positive correlation between normal triglyceride concentration and T2DM, and the triglyceride concentration increased without a threshold effect.

### 3.5 Subgroup analyses

**Figure 4:**
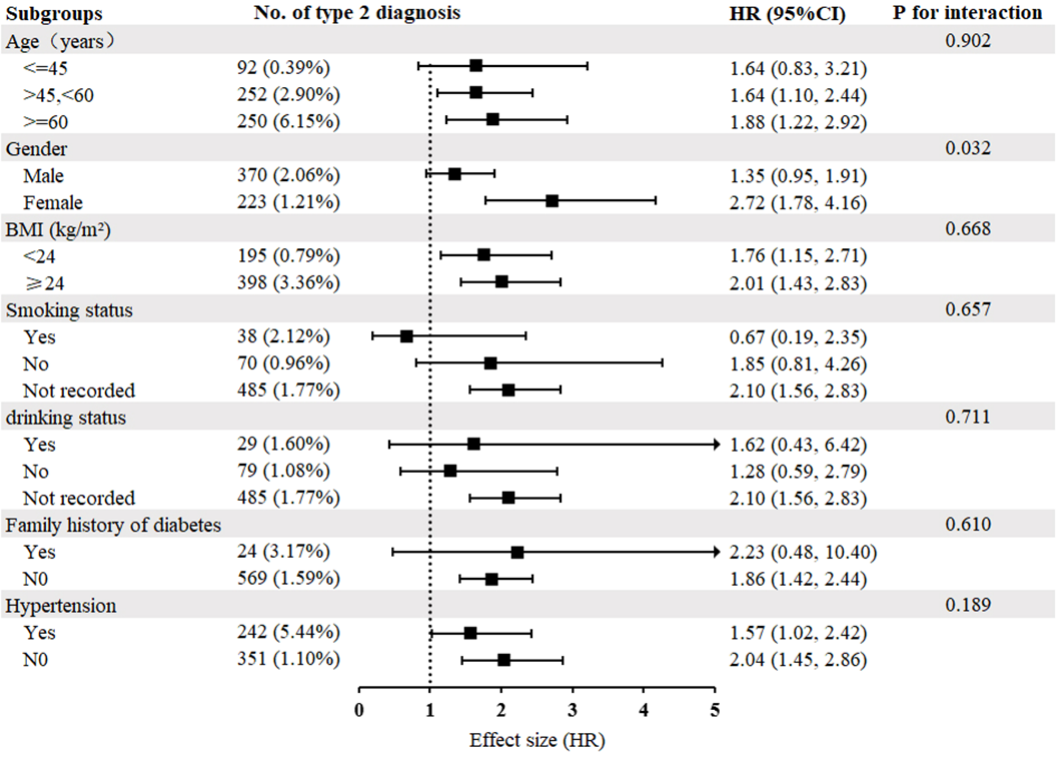
Subgroup analysis of the relationship between triglyceride concentration and diabetes mellitus type 2. Adjustments were made based on age, sex, BMI, SBP, DBP, FPG, cholesterol, HDL-C, LDL-C, ALT, AST, BUN, CCR, hypertension, smoking status, drinking status, family history of diabetes, except for subgroup variables. As in **Table 1**.

**Figure 4** illustrates the findings of the subgroup analysis performed to examine the association between triglyceride concentration and the development of T2DM. Participants were stratified by age, sex, BMI, smoking status, drinking status, hypertension, and family history of diabetes to explore other potential risk factors and identify any special populations that may affect this relationship. Subgroup analyses revealed no significant interactions between triglyceride concentration and the incidence of T2DM across various strata of age, BMI, smoking status, drinking status, hypertension, and family history of diabetes. However, sex was observed to modify the relationship between triglyceride concentration and incidence of T2DM (P for interaction<0.05); females exhibited a more pronounced association [HR=2.72, 95% CI (1.78, 4.16)], whereas males showed a less significant association [HR=1.35, 95% CI (0.95, 1.91)].

## 4 Discussion

The findings of this extensive longitudinal cohort study conducted in a Chinese adult population suggest that even among individuals considered healthy within the general population, an increase in triglyceride levels within the normal range is linked to elevated susceptibility to T2DM. After adjusting for covariates, the results indicate that for every 1 mmol/L increase in normal triglyceride levels, there was an 81% increase in the risk of developing T2DM. Furthermore, our analysis revealed a consistent and proportional relationship between normal triglyceride levels and the risk of developing T2DM, spanning the entire spectrum of normal triglyceride values. According to our subgroup analysis, sex was a significant factor in determining normal triglyceride concentration and the risk of developing T2DM. Specifically, in the female cohort, a stronger positive correlation was observed between normal triglyceride levels and the risk of T2DM.

Increasing evidence suggests that triglycerides, when combined with other indicators, serve as independent predictive factors for the risk of developing T2DM. A longitudinal study spanning 5 years and comprising 25,248 participants from China discovered that the triglyceride-glucose index (TyG index) shows potential as a reliable marker for forecasting the transition from normal blood sugar levels to prediabetes among the Chinese population (16). In a 13-year cohort study conducted in Japan with a participant pool of 15,464 individuals, multivariate Cox regression analysis revealed the triglyceride-glucose BMI index (TyG-BMI) as an autonomous prognosticator for the initiation of T2DM (10). A common thread among these studies is the use of triglycerides as a fundamental parameter, indicating a potential association between triglyceride levels and diabetes. This collective observation underscores the possibility of an underlying connection between triglyceride concentration and the development of diabetes.

Previous studies have also indicated a positive correlation between elevated triglyceride levels and the risk of diabetes. In, 2019, Zhao et al. conducted an 8-year follow-up study in China involving 7,241 middle-aged and senior individuals with a mean baseline age of 61.49 ± 13.85 years. The study showed that compared to the normal triglyceride group (TG<1.7 mmol/L), the hazard ratios for the critical group (TG=1.7–2.25 mmol/L) and high triglyceride group (TG>2.26 mmol/L) were 1.30 (95% CI 1.04–1.62) and 1.54 (95% CI 1.24–1.90), respectively (8). In, 2018, Zheng et al. conducted a study on the relationship between triglyceride levels and glycemic control in patients with T2DM who were undergoing insulin therapy (17). This study demonstrated that inhibiting triglyceride levels can lead to optimized glycemic control in patients with T2DM. An increasing body of literature has established a positive correlation between elevated triglyceride levels and the risk of developing T2DM. The interaction between high levels of triglycerides and glucose may lead to “glucolipotoxicity” exerting negative effects on the function and quantity of beta cells. This phenomenon is mediated through multiple pathways and is influenced by various factors (18). Elevated triglyceride levels may result in insulin resistance, which is a significant risk factor for diabetes. During the development of insulin resistance and diabetes, beta cells undergo a series of adaptations and failures. In the early stage of insulin resistance, beta cells compensate through increased function and quantity (referred to as glucolipid adaptation). However, over time, sustained hyperglycemia and lipid levels can lead to gradual deterioration of beta cell function, ultimately leading to the development of diabetes (19).

To our knowledge, few studies have investigated the relationship between normal fasting triglyceride levels and the incidence of T2DM in the general population. In, 2022, Szili-Torok et al. conducted a median follow-up of 11.4 years on 2,085 participants with normal triglyceride levels and no metabolic syndrome in the Netherlands. This study showed an independent correlation between the incidence of T2DM and triglyceride levels within the normal range (20). In their investigation, the researchers observed a consistent association between normal triglyceride levels and the risk of T2DM across various subgroups, including age, BMI, smoking status, waist circumference, hypertension, glomerular filtration rate, and antihypertensive drug use. Similarly, we conducted a subgroup analysis and revealed a stable relationship between triglyceride levels and T2DM risk according to age, BMI, smoking status, drinking status, family history of diabetes, and hypertension. However, within the sex subgroup, we observed a stronger correlation between normal triglyceride levels and the risk of T2DM in females than in males. Numerous sex-based differences have been observed in the occurrence and progression of diabetes (21). Moreover, similar sex differences have been reported to exist in the relationship between body roundness index (BRI) and T2DM (22). These differences may be associated with the protective effects of estrogen. Before menopause, women exhibit a lower incidence of insulin resistance and T2DM than men of similar age. However, this protective effect is weakened or disappeared after menopause, and men and postmenopausal women showed similar incidences of insulin resistance and T2DM (23–25). Insulin resistance and the incidence of T2DM are also influenced by metabolic differences between the sexes (26). Previous studies have shown that women have lower insulin sensitivity than men of the same age, which may make women more vulnerable to insulin resistance, thus increasing the risk of diabetes (27). In addition, women tend to have fat distribution in the waist and abdomen after postpartum and menopause, which is closely related to the increased risk of metabolic syndrome and diabetes (28). Additionally, TyG is calculated by multiplying fasting triglyceride levels and fasting blood sugar concentrations; therefore, as the value of the TyG index increases, the severity of insulin resistance also intensifies (29, 30). Sex differences in the association between normal triglyceride levels and the risk of developing T2DM could potentially be explained by these factors. Owing to the current lack of relevant data to define menopause and insulin resistance, our study was unable to delve deeper into the specific role of normal triglyceride levels in sex differences and T2DM. Further research is required for a more comprehensive analysis.

This study has important clinical implications, suggesting that re-evaluating the normal range of plasma triglycerides may be necessary. Our results suggest that individuals can benefit from adopting certain lifestyle modifications, including reducing alcohol consumption, increasing physical activity, substituting saturated fats with monounsaturated fats (such as extra virgin olive oil) and polyunsaturated fats (non-tropical plant oils), and limiting the intake of refined carbohydrate-rich foods such as sucrose and fructose (31). By minimizing triglyceride levels to the greatest extent possible, these guidelines can mitigate triglyceride levels and attenuate the probability of T2DM onset in a broader population. However, to ensure the generalizability of our findings, it is essential to obtain independent validation from other populations, including diverse ethnicities and socioeconomic backgrounds. Furthermore, it would be highly valuable to conduct experimental studies in humans and appropriate preclinical models to elucidate the underlying mechanisms by which triglycerides affect insulin resistance and beta-cell function. Our findings provide a foundation for further investigations into the role of triglycerides in T2DM pathogenesis, potentially leading to the development of novel therapeutic strategies.

Our study has several strengths. First, this was a large-scale population-based cohort study that enabled us to observe the correlation between normal triglyceride levels and the risk of T2DM. However, further research on the pathological and physiological mechanisms is needed to strengthen this association and overcome potential limitations. Second, we implemented rigorous statistical adjustments to minimize the influence of confounding factors. We systematically included all the relevant covariates in the Cox regression model and compared the regression coefficients by inputting or eliminating each covariate individually in the basic and complete models. Third, our study is the most comprehensive investigation of the association between normal triglyceride levels and the risk of T2DM, with a substantial sample size and extensive adjustments for confounding factors. Fourth, we conducted a sensitivity analysis to ensure the robustness of our findings across subgroups. Moreover, we employed curve function fitting to reveal a persistent linear relationship between normal triglyceride concentration and the risk of T2DM. However, our study has a few limitations. First, owing to data constraints, we could not obtain information on glycosylated hemoglobin, a valuable biomarker for diabetes diagnosis. Second, more detailed follow-up data such as sleep, nutrition, and exercise status were unavailable, potentially introducing additional confounding factors that should be considered. Third, the incidence of T2DM may have been underestimated, as oral glucose tolerance tests were not performed. Finally, our study focused on a Chinese population cohort, primarily from urban areas in southern China; thus, the generalizability of our results to northern Chinese and non-Chinese populations requires further investigation.

## 5 Conclusion

Our research demonstrates an independent positive association between normal triglyceride levels and the likelihood of developing diabetes mellitus type 2. For every 1 mmol/L increase in triglycerides, the risk of developing T2DM increased by 81%. Moreover, we established a positive linear relationship between triglyceride levels and the risk of T2DM across the entire range of normal triglyceride concentrations. Further investigation is necessary to elucidate the causal relationship between normal triglyceride levels and T2MD.

## Data availability statement

Publicly available datasets were analyzed in this study. This data can be found here: http://charls.pku.edu.cn/zh-CN.

## Ethics statement

The studies involving humans were approved by Rich Healthcare Group Review Board. The studies were conducted in accordance with the local legislation and institutional requirements. Written informed consent for participation was not required from the participants or the participants’ legal guardians/next of kin because For this type of study, formal consent is not required.

## Author contributions

RG: Data curation, Writing – original draft, Writing – review & editing, Methodology, Project administration, Software. LW: Supervision, Writing – review & editing. YC: Supervision, Writing – review & editing. WZ: Funding acquisition, Methodology, Supervision, Writing – review & editing, Project administration.

## References

[1] Sun H Saeedi P Karuranga S Pinkepank M Ogurtsova K Duncan BB. IDF Diabetes Atlas: Global, regional and country-level diabetes prevalence estimates for 2021 and projections for 2045. Diabetes Res Clin practice. (2022) 183:109119. doi: 10.1016/j.diabres.2021.109119 PMC11057359 34879977

[2] Ogurtsova K Guariguata L Barengo NC Ruiz PL Sacre JW Karuranga S. IDF diabetes Atlas: Global estimates of undiagnosed diabetes in adults for 2021. Diabetes Res Clin practice. (2022) 183:109118. doi: 10.1016/j.diabres.2021.109118 34883189

[3] Reiner Ž. Hypertriglyceridaemia and risk of coronary artery disease. Nat Rev Cardiol (2017) 14(7):401–11. doi: 10.1038/nrcardio.2017.31 28300080

[4] Halldin AK Lissner L Lernfelt B Björkelund C. Cholesterol and triglyceride levels in midlife and risk of heart failure in women, a longitudinal study: The prospective population study of women in Gothenburg. BMJ Open (2020) 10(6):e036709. doi: 10.1136/bmjopen-2019-036709 PMC7279659 32503873

[5] Peng J Luo F Ruan G Peng R Li X. Hypertriglyceridemia and atherosclerosis. Lipids Health disease. (2017) 16(1):233. doi: 10.1186/s12944-017-0625-0 PMC5719571 29212549

[6] Wang C Zhao Z Deng X Cai Z Gu T Li L. Association of triglyceride-glucose with cardiac hemodynamics in type 2 diabetes. Diabetes Vasc Dis Res (2022) 19(1):14791641221083396. doi: 10.1177/14791641221083396 PMC8972936 35345912

[7] Liu L Wu Z Zhuang Y Zhang Y Cui H Lu F. Association of triglyceride-glucose index and traditional risk factors with cardiovascular disease among non-diabetic population: A 10-year prospective cohort study. Cardiovasc diabetology. (2022) 21(1):256. doi: 10.1186/s12933-022-01694-3 PMC9700958 36434636

[8] Zhao J Zhang Y Wei F Song J Cao Z Chen C. Triglyceride is an independent predictor of type 2 diabetes among middle-aged and older adults: a prospective study with 8-year follow-ups in two cohorts. J Trans Med (2019) 17(1):403. doi: 10.1186/s12967-019-02156-3 PMC6894231 31801571

[9] Peng J Zhao F Yang X Pan X Xin J Wu M. Association between dyslipidemia and risk of type 2 diabetes mellitus in middle-aged and older Chinese adults: a secondary analysis of a nationwide cohort. BMJ Open (2021) 11(5):e042821. doi: 10.1136/bmjopen-2020-042821 PMC8154929 34035089

[10] Song B Zhao X Yao T Lu W Zhang H Liu T. Triglyceride glucose-body mass index and risk of incident type 2 diabetes mellitus in Japanese people with normal glycemic level: A population-based longitudinal cohort study. Front endocrinology. (2022) 13:907973. doi: 10.3389/fendo.2022.907973 PMC9336540 35909552

[11] Zou S Yang C Shen R Wei X Gong J Pan Y. Association between the triglyceride-glucose index and the incidence of diabetes in people with different phenotypes of obesity: A retrospective study. Front endocrinology. (2021) 12:784616. doi: 10.3389/fendo.2021.784616 PMC8695922 34956095

[12] Kim JA Kim J Roh E Hong SH Lee YB Baik SH. Triglyceride and glucose index and the risk of gestational diabetes mellitus: A nationwide population-based cohort study. Diabetes Res Clin practice. (2021) 171:108533. doi: 10.1016/j.diabres.2020.108533 33157117

[13] Vesković M Šutulović N Hrnčić D Stanojlović O Macut D Mladenović D. The interconnection between hepatic insulin resistance and metabolic dysfunction-associated steatotic liver disease-the transition from an adipocentric to liver-centric approach. Curr Issues Mol Biol (2023) 45(11):9084–102. doi: 10.3390/cimb45110570 PMC10670061 37998747

[14] Chen Y Zhang XP Yuan J Cai B Wang XL Wu XL. Association of body mass index and age with incident diabetes in Chinese adults: A population-based cohort study. BMJ Open (2018) 8(9):e021768. doi: 10.1136/bmjopen-2018-021768 PMC6169758 30269064

[15] 4. Comprehensive medical evaluation and assessment of comorbidities: Standards of medical care in diabetes-2021. Diabetes Care (2021) 44(Suppl 1):S40–s52. doi: 10.2337/dc21-S004 33298415

[16] Chen X Liu D He W Hu H Wang W. Predictive performance of triglyceride glucose index (TyG index) to identify glucose status conversion: A 5-year longitudinal cohort study in Chinese pre-diabetes people. J Trans Med (2023) 21(1):624. doi: 10.1186/s12967-023-04402-1 PMC10503019 37715242

[17] Zheng D Dou J Liu G Pan Y Yan Y Liu F. Association between triglyceride level and glycemic control among insulin-treated patients with type 2 diabetes. J Clin Endocrinol Metab (2019) 104(4):1211–20. doi: 10.1210/jc.2018-01656 30418583

[18] Vilas-Boas EA Almeida DC Roma LP Ortis F Carpinelli AR. Lipotoxicity and β-cell failure in type 2 diabetes: Oxidative stress linked to NADPH oxidase and ER stress. Cells. (2021) 10(12):3328. doi: 10.3390/cells10123328 34943836 PMC8699655

[19] Plötz T von Hanstein AS Krümmel B Laporte A Mehmeti I Lenzen S. Structure-toxicity relationships of saturated and unsaturated free fatty acids for elucidating the lipotoxic effects in human EndoC-βH1 beta-cells. Biochim Biophys Acta Mol basis disease. (2019) 1865(11):165525. doi: 10.1016/j.bbadis.2019.08.001 31398470

[20] Szili-Torok T Bakker SJL Tietge UJF. Normal fasting triglyceride levels and incident type 2 diabetes in the general population. Cardiovasc diabetology. (2022) 21(1):111. doi: 10.1186/s12933-022-01530-8 PMC9206357 35717188

[21] Greenhill C. Obesity: Sex differences in insulin resistance. Nat Rev Endocrinology. (2018) 14(2):65. doi: 10.1038/nrendo.2017.168 29219148

[22] Zhao W Tong J Li J Cao Y. Relationship between body roundness index and risk of type 2 diabetes in Japanese men and women: A reanalysis of a cohort study. Int J endocrinology. (2021) 2021:4535983. doi: 10.1155/2021/4535983 35003255 PMC8731295

[23] Kautzky-Willer A Leutner M Harreiter J. Sex differences in type 2 diabetes. Diabetologia. (2023) 66(6):986–1002. doi: 10.1007/s00125-023-05891-x 36897358 PMC10163139

[24] Tramunt B Smati S Grandgeorge N Lenfant F Arnal JF Montagner A. Sex differences in metabolic regulation and diabetes susceptibility. Diabetologia. (2020) 63(3):453–61. doi: 10.1007/s00125-019-05040-3 PMC6997275 31754750

[25] Kautzky-Willer A Harreiter J Pacini G. Sex and gender differences in risk, pathophysiology and complications of type 2 diabetes mellitus. Endocrine Rev (2016) 37(3):278–316. doi: 10.1210/er.2015-1137 27159875 PMC4890267

[26] Ciarambino T Crispino P Leto G Mastrolorenzo E Para O Giordano M. Influence of gender in diabetes mellitus and its complication. Int J Mol Sci (2022) 23(16):8850 doi: 10.3390/ijms23168850 36012115 PMC9408508

[27] Xie Q Kuang M Lu S Huang X Wang C Zhang S. Association between MetS-IR and prediabetes risk and sex differences: a cohort study based on the Chinese population. Front endocrinology. (2023) 14:1175988. doi: 10.3389/fendo.2023.1175988 PMC10226663 37255977

[28] Hadaegh F Abdi A Kohansal K Hadaegh P Azizi F Tohidi M. Gender differences in the impact of 3-year status changes of metabolic syndrome and its components on incident type 2 diabetes mellitus: A decade of follow-up in the Tehran Lipid and Glucose Study. Front endocrinology. (2023) 14:1164771. doi: 10.3389/fendo.2023.1164771 PMC10248400 37305040

[29] Tahapary DL Pratisthita LB Fitri NA Marcella C Wafa S Kurniawan F. Challenges in the diagnosis of insulin resistance: Focusing on the role of HOMA-IR and Tryglyceride/glucose index. Diabetes Metab syndrome. (2022) 16(8):102581. doi: 10.1016/j.dsx.2022.102581 35939943

[30] Ramdas Nayak VK Satheesh P Shenoy MT Kalra S. Triglyceride Glucose (TyG) Index: A surrogate biomarker of insulin resistance. JPMA J Pakistan Med Assoc (2022) 72(5):986–8. doi: 10.47391/jpma.22-63 35713073

[31] Laufs U Parhofer KG Ginsberg HN Hegele RA. Clinical review on triglycerides. Eur Heart J (2020) 41(1):99–109c. doi: 10.1093/eurheartj/ehz785 31764986 PMC6938588

